# Evolution of a *cis*-Acting SNP That Controls Type VI Secretion in Vibrio cholerae

**DOI:** 10.1128/mbio.00422-22

**Published:** 2022-05-23

**Authors:** Siu Lung Ng, Sophia Kammann, Gabi Steinbach, Tobias Hoffmann, Peter J. Yunker, Brian K. Hammer

**Affiliations:** a School of Biological Sciences, Georgia Institute of Technology, Atlanta, Georgia, USA; b School of Physics, Georgia Institute of Technology, Atlanta, Georgia, USA; c Parker H. Petit Institute for Bioengineering & Bioscience, Georgia Institute of Technology, Atlanta, Georgia, USA; d Center for Microbial Diseases and Infection, Georgia Institute of Technology, Atlanta, Georgia, USA; National Institute of Child Health and Human Development (NICHD)

**Keywords:** vibrio cholerae, evolution, gene regulation, secretion systems, signal transduction, transcription factors

## Abstract

Mutations in regulatory mechanisms that control gene expression contribute to phenotypic diversity and thus facilitate the adaptation of microbes and other organisms to new niches. Comparative genomics can be used to infer rewiring of regulatory architecture based on large effect mutations like loss or acquisition of transcription factors but may be insufficient to identify small changes in noncoding, intergenic DNA sequence of regulatory elements that drive phenotypic divergence. In human-derived Vibrio cholerae, the response to distinct chemical cues triggers production of multiple transcription factors that can regulate the type VI secretion system (T6), a broadly distributed weapon for interbacterial competition. However, to date, the signaling network remains poorly understood because no regulatory element has been identified for the major T6 locus. Here we identify a conserved *cis*-acting single nucleotide polymorphism (SNP) controlling T6 transcription and activity. Sequence alignment of the T6 regulatory region from diverse V. cholerae strains revealed conservation of the SNP that we rewired to interconvert V. cholerae T6 activity between chitin-inducible and constitutive states. This study supports a model of pathogen evolution through a noncoding *cis*-regulatory mutation and preexisting, active transcription factors that confers a different fitness advantage to tightly regulated strains inside a human host and unfettered strains adapted to environmental niches.

## INTRODUCTION

A central role in the dynamic, temporal control of gene expression is played by transcription factors (TFs), diffusible “*trans*” products that bind to molecular switches within DNA sequences termed “*cis*”-regulatory elements (CREs). In eukaryotes, where horizontal gene transfer (HGT) is rare, mutations in CREs that alter TF binding sites are major contributors to phenotypic diversity (1–3). In bacteria, pervasive HGT can alter entire regulatory circuits that allow adaptation to new niches, as prominently demonstrated in Vibrio fischeri, where host range is altered by the presence or absence of a histidine kinase RcsS, which regulates biofilm and colonization genes via indirect mechanisms (4, 5). By contrast, specific mutations at CREs in noncoding DNA are more difficult to identify and receive less attention as drivers of phenotypic divergence and evolutionary adaptation (6). Thus, elucidation of how microbes adapt to new niches, a process of fundamental importance in bacterial pathogenesis, requires coupling of genome-wide computational methods with experimental approaches to map the *cis-* and *trans*-regulatory interactions across and within species.

To understand how mutations play a role in microbial adaptation, pathogenic viruses and bacteria with lifestyles that exploit niches within and outside a human host are of great interest. Following ingestion, pandemic strains of the bacterium Vibrio cholerae can colonize the human gastrointestinal tract and secrete the cholera toxin that leads to the often fatal diarrhea responsible for seven pandemics to date (7–9). Conversely, V. cholerae isolated from nonhuman niches lack the horizontally acquired prophage that carries the cholera toxin, and cause mild illness (10). By contrast, all sequenced V. cholerae encode a type VI secretion system (T6), a broadly distributed “nano-harpoon” weapon that injects toxic effector proteins into neighboring bacterial cells, leading to cell envelope damage and cell lysis (11, 12). Due to its broad distribution among bacteria including those of the human gut, there is intense interest in understanding the T6 interactions between our microbiota and foreign pathogens, and whether they can be manipulated to influence health (13).

V. cholerae obtained from humans carry a limited arsenal of effectors and a T6 believed to be tailored for *in vivo* success (11, 14–19), while strains from nonhuman niches encode a more diverse effector repertoire (11, 14, 20, 21). To date, however, adaptative evolution mechanisms of T6 regulation in V. cholerae derived from nonhuman sources have largely been overlooked. Since the discovery of T6, studies of human-derived strains identify two primary TFs for T6 activation (22–26). T6 control in pandemic strains (e.g., C6706 and A1552) requires QstR, which is positively regulated by multiple external cues, including chitin that triggers TfoX production, and quorum-sensing autoinducers that control the well-studied LuxO/HapR regulatory circuit (27–30). QstR also contains a C-terminal DNA binding domain postulated to interact with a presumptive CRE of the major T6 gene cluster, yet how QstR-DNA interaction affects T6 transcription remains unclear (23, 27). On the other hand, T6 regulation in nonpandemic strain V52, which causes mild disease, requires TfoY. Expression of *tfoY* is modulatable by the intracellular second messenger 3′,5′-cyclic diguanylic acid (c-di-GMP) (25, 26). At low c-di-GMP levels, *tfoY* expression is posttranscriptionally regulated by a *cis*-acting riboswitch located upstream of the gene. At high c-di-GMP levels *tfoY* is regulated by transcription factor VpsR, which binds the second messenger (31). Despite significant progress over the past decade in uncovering the signaling systems that modulate QstR and TfoY, the mechanisms by which these two regulatory proteins control gene expression remain unclear. Similarly, direct regulators of T6 transcription, still remain elusive, with only one putative T6 CRE described (23). Elucidation of the differences in intraspecies T6 regulatory mechanisms between diverse V. cholerae isolates will provide insights into how pathogens emerge from nonpathogenic progenitors.

To understand the regulatory differences in V. cholerae strains, we examine several environmental isolates that exhibit T6-mediated killing (32). Despite encoding functional signaling circuity and TFs, we find that QstR is dispensable for killing and that TfoY plays only a minor role for killing in the strains tested. Thus, existing regulatory models fail to explain the T6 control in V. cholerae from human and nonhuman sources. Genomic analysis identifies one conserved noncoding single-nucleotide polymorphism (SNP) that we show interconverts V. cholerae T6 activity between chitin-inducible and constitutive states, which are QstR-dependent and TfoY-independent, respectively. We demonstrate that noncoding SNPs can rewire *cis*-regulatory elements, which may aid in adaptation of bacteria to different niches, including the human host.

## RESULTS AND DISCUSSION

### Constitutive, *in vitro* T6 activity requires neither QstR nor TfoY in many environmental V. cholerae isolates.

**FIG 1:**
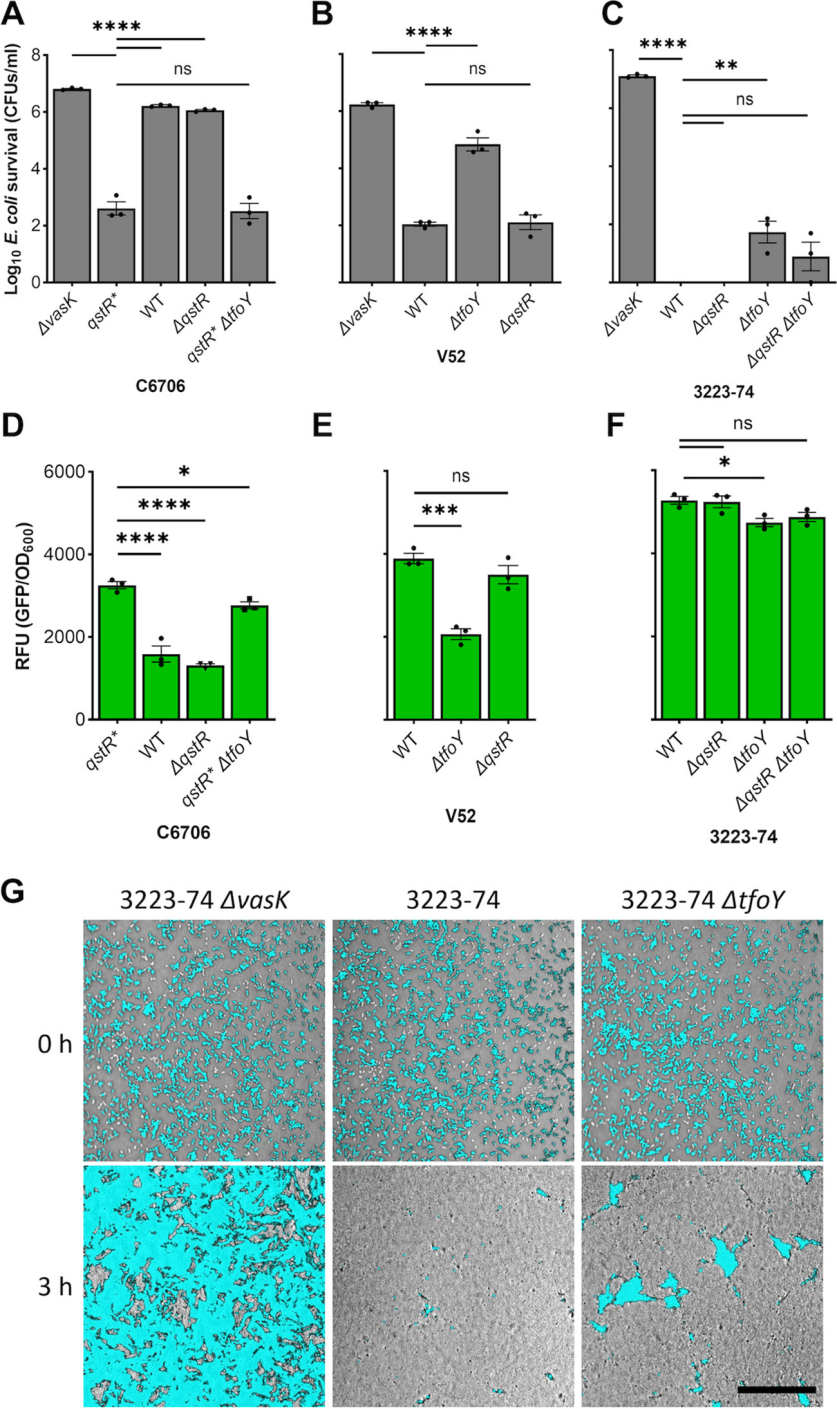
Vibrio cholerae 3223-74 T6 activity is QstR- and TfoY-independent. (A to C) V. cholerae strains with the indicated genotypes were cocultured with chloramphenicol resistant (Cm^r^) E. coli followed by determination of E. coli survival by counting of CFU on LB agar with Cm. A V. cholerae *ΔvasK* mutant defective in T6 assembly served as a T6- negative control. (D to F) Relative Fluorescence Units are from reporters with *gfp* fused to the intergenic region 5′ of *vipA* derived from the strains shown. The mean value ± S.E. from cocultures (A to C) and monocultures (D to F) are derived from three independent biological replicates. A one-way ANOVA with Dunnett *post hoc* test was conducted to determine the significance: ns denotes not significant, ****, *P* ≤ 0.0001; ***, *P* ≤ 0.001; **, *P* ≤ 0.01; *, *P* ≤ 0.05. (G) E. coli cells expressing constitutive *gfp* were competed against 3223-74, with the same frame imaged at 0 h and 3 h by confocal microscopy. In the images, *gfp* signal from the E. coli is overlaid on top of bright-light images of the coculture. Scale bar = 50 μm.

In pandemic C6706, high cell density conditions (HCD) and chitin are required for induction of *qstR* which leads to activation of T6 genes. In the absence of chitin, C6706 with *qstR* expressed from a heterologous promoter (defined here as qstR*) reduces survival of Escherichia coli “target” cells in coculture by over 4-orders of magnitude (~10,000), compared with wildtype (WT) C6706, a T6 strain with a mutation in an essential structural gene (*ΔvasK*), and a strain with a *ΔqstR* mutation (Fig. 1A) (29). Deletion of *tfoY* does not reduce the killing activity of the T6^+^ qstR* strain, but eliminates the robust killing in the nonpandemic strain V52 (serogroup O37), which requires TfoY but not QstR (Fig. 1B) (26).

To determine whether QstR or TfoY participates in control of the T6 in nonhuman derived strains, we examined 3223-74, a genetically amenable, T6-proficient environmental strain (32). Like V52, 3223-74 does not require QstR to efficiently kill E. coli in conditions without chitin, but surprisingly, also does not require TfoY. Isogenic strains carrying the *ΔtfoY* and *ΔqstR ΔtfoY* mutations retain >99.99% killing activity, with only modest E. coli survival (Fig. 1C). Gene fusions of the 5′ intergenic region (IGR) of the major T6 cluster of each strain fused to green fluorescent protein (*gfp*) confirm that transcriptional differences account for the killing observed, with maximal *gfp* expression mirroring activity (i.e., low E. coli survival with high *gfp* expression, and *vice versa*) (Fig. 1D to F). To confirm that expression of the major T6 loci is not influenced by transcriptional read-through from a regulatory element upstream of the IGR, a T7 terminator (33) was inserted directly after the stop codon of *vca0106* in V. cholerae with activated T6 (Fig. S1). We observed no differences in T6 killing, demonstrating that the IGR is sufficient for control of the major T6 locus. Confocal microscopy reinforces the negligible role of TfoY on killing by 3223-74, with a Δ*tfoY* mutation having little effect on killing WT (Fig. 1G). Transcription of plasmid-borne reporters is significantly higher in V. cholerae than in E. coli (Fig. S2), supporting a hypothesis that an additional V. cholerae-specific regulator of the T6 may remain to be identified.

10.1128/mbio.00422-22.1 FIG S1 Activity of the major T6 gene cluster is not controlled by transcriptional read-through. (A) Schematic shows the wildtype T6 5’ IGR. The T7 terminator DNA sequence (33) encoding an RNA hairpin (underlined) is indicted, as well as the location the terminator was inserted before and after the T6 5’ IGR. (B) Competition assays were conducted by co-culturing V. cholerae and Cm^r^ E. coli target followed by determination of E. coli survival by counting of CFUs on LB agar with Cm. The V. cholerae *ΔvasK* mutant served as a T6- negative control. Data shown are mean values ± S.E. from three independent biological replicates. A one-way ANOVA with Dunnett post-hoc test was conducted to determine the significance. ns, not significant; ****, *P* ≤ 0.0001; ns > 0.05. Download FIG S1, TIF file, 1.3 MB. Copyright © 2022 Ng et al. 2022 Ng et al. https://creativecommons.org/licenses/by/4.0/ This content is distributed under the terms of the Creative Commons Attribution 4.0 International license.

10.1128/mbio.00422-22.2 FIG S2 The major V. cholerae T6 promoter is not constitutively expressed in E. coli. V. cholerae or E. coli carrying a plasmid-encoded *gfp* gene driven by either the C6706 or 3223-74 5’ T6 IGR was grown in liquid LB with Cm. *gfp* is represented as relative fluorescent units per OD_600_ (RFU). Data shown are mean values ± S.E. from 3 independent biological replicates. A one-way ANOVA with Tukey post-hoc test was conducted to determine the significance: ****, *P* ≤ 0.0001; ***, *P* ≤ 0.001. Download FIG S2, TIF file, 1.2 MB. Copyright © 2022 Ng et al. 2022 Ng et al. https://creativecommons.org/licenses/by/4.0/ This content is distributed under the terms of the Creative Commons Attribution 4.0 International license.

**FIG 2:**
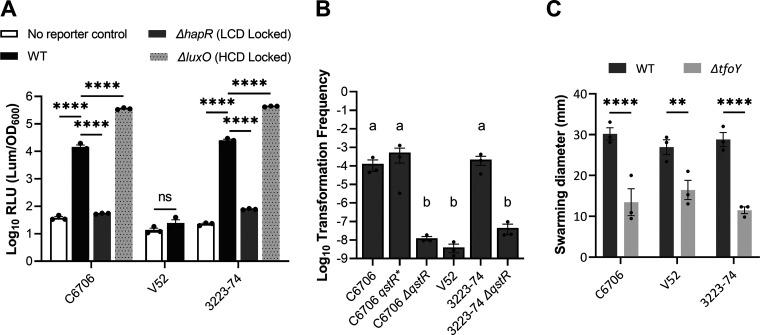
Vibrio cholerae 3223-74 encodes functional HapR, QstR, and TfoY. (A) V. cholerae strains with and without a QS-dependent *lux* reporter cosmid (pBB1) were grown in liquid LB with relative luminescence units per OD_600_ measured at HCD (OD_600_ = 0.6-0.8). Statistical analyses were conducted with one-way ANOVA with Tukey *post hoc* test (C6706 and 3223-74) and one-tailed Student’s *t* test (V52). The *ΔhapR* mutant is defective at QS and effectively “locked” at low cell density, while the *ΔluxO* mutant that constitutively produces HapR is effectively “locked” at high cell density. (B) V. cholerae strains with the indicated genotypes were grown in ASW with crab shell and exogenous Spec-marked genomic DNA. Transformation frequency = Spec^r^ CFU mL^−1^/total CFU mL^−1^. Statistical analyses were conducted with one-way ANOVA with Tukey *post hoc* test. Letters “a” and “b” identify statistically significance (*P* ≤ 0.05) of transformation frequency between V. cholerae strains. (C) V. cholerae strains were inoculated on 0.3% LB agar and grew overnight. Statistical analyses were conducted with one-tailed Student’s *t* test. Colony diameters were physically measured from the furthest edges. All data shown are the mean ± S.E. from 3 independent biological replicates. ns: not significant, ****, *P* ≤ 0.0001; **, *P* ≤ 0.01.

To probe each strain’s T6-related regulatory circuitry, we measured canonical behaviors under the control of HapR, QstR, and TfoY; quorum sensing (QS) controlled bioluminescence, natural transformation, and motility, respectively (31, 34, 35). As expected, each TF is intact in C6706; but like several V. cholerae strains, V52 lacks a functional *hapR* gene that prevents QS and natural transformation (36, 37). Nonetheless, V52 encodes a functional *tfoY* that controls motility (Fig. 2A and B) (26). Interestingly, the regulatory circuity of V. cholerae 3223-74 is intact, like C6706, confirming that it encodes functional TFs (Fig. 2C), which are nonetheless expendable for T6-mediated killing. Nucleoid associated proteins (NAPs) that bind DNA both specifically and nonspecifically (39) may contribute to T6 transcription. NAPs participate in regulation of many promoters in numerous bacteria including *Vibrios* (40), yet NAP regulation and expression levels may differ in C6706 and 3223-74 (41). It is also possible that T6 regulation is complex and involves more than one TF specific to V. cholerae.

### A SNP in the T6 intergenic region confers QstR-dependency.

**FIG 3:**
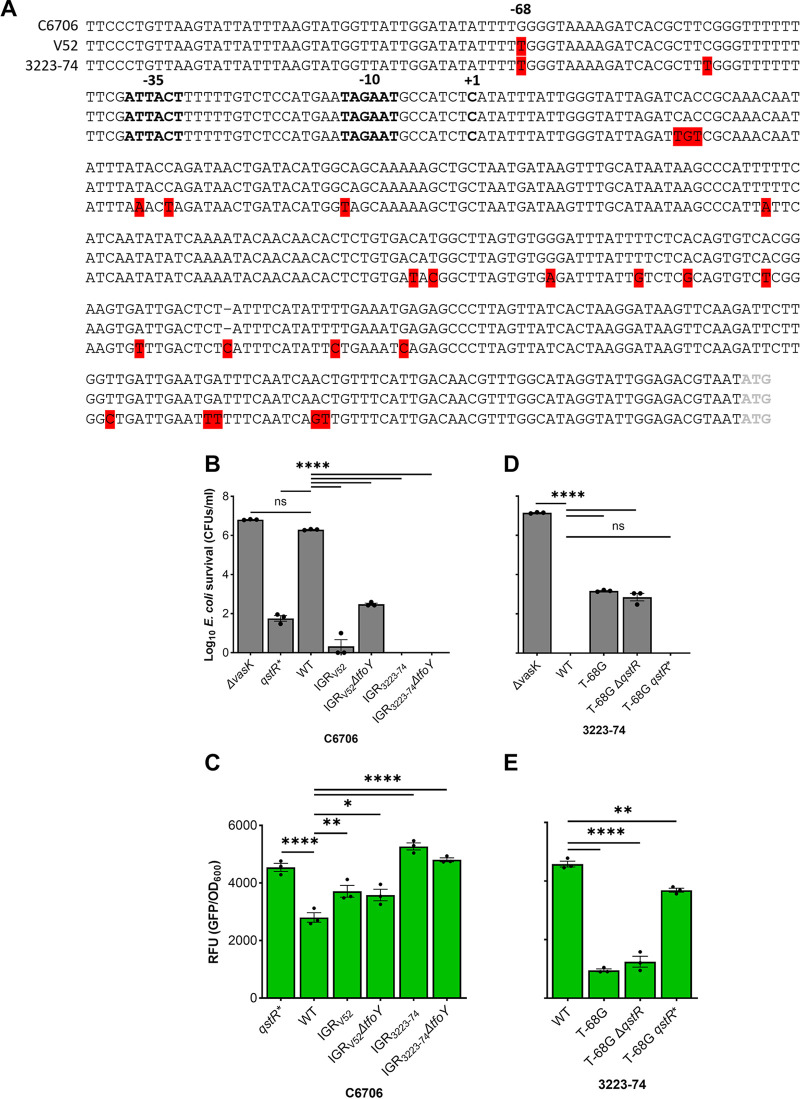
G-68T mutation abolishes QstR dependence in C6706 and T-68G confers QstR dependence to 3223-74. (A) Alignment of the IGR upstream of *vipA* was conducted using MUSCLE. SNPs and MNPs are highlighted in red, one gap indicated with a “–,” the putative promoter and the transcriptional start site (TSS; +1) in bold, and the start codon of *vipA* in gray. (B) the C6706 5′ IGR of *vipA* was replaced with the IGR from either V52 or 3223-74. (D) A T-68G mutation in the 5′ IGR of *vipA* was introduced into 3223-74 with different *qstR* alleles. Competition assays were conducted by coculturing V. cholerae killers and Cm^r^ E. coli target followed by determination of E. coli survival by counting of CFU (CFU) on LB agar with Cm. The V. cholerae *ΔvasK* mutant unable to assemble a functional T6 served as a T6- negative control. (C, E) Shown are fluorescence levels of transcriptional reporters with *gfp* fused to corresponding IGRs of *vipA* expressed in either C6706 (C) or 3223-74 (E). Shown are mean values ± S.E. from three independent biological replicates of cocultures (B and D) and monocultures (C and E). A one-way ANOVA with Dunnett *post hoc* test was conducted to determine the significance. ns, not significant; ****, *P* ≤ 0.0001; **, *P* ≤ 0.01; *, *P* ≤ 0.05.

Human and environmental isolates of V. cholerae we have characterized prior (32) share ≥97% average nucleotide identity with many chromosomal differences (11). Yet, inspection of the T6 IGRs of C6706, V52, and 3223-74 revealed only 17 SNPs and three multinucleotide polymorphisms (Fig. 3A), which we hypothesized could contribute to the differences in T6 transcription and killing activity observed. To address this, we replaced the T6 IGR of C6706 on the chromosome with that from V52 and 3223-74 and measured killing activity. While C6706 carrying the qstR* allele, but not WT, adeptly kills E. coli, both IGR replacements increase the killing efficiency of WT C6706 by 5- to 6-orders of magnitude (Fig. 3B), mimicking the robust killing observed by WT V52 and 3223-74 (Fig. 1B and C). Deletion of *tfoY* but not *qstR* in C6706 with V52’s IGR increases E. coli survival (~ 2-logs), as observed with V52, but does not alter E. coli survival with 3223-74’s IGR (Fig. 3B; Fig. S3). Chromosomal transcriptional *gfp* reporters with identical mutations were elevated relative to WT C6706 in each IGR replacement strain (Fig. 3C), consistent with the enhanced killing detected. How TfoY controls gene expression is currently unknown and beyond the scope here. However, we speculate that slight differences detected in survival but not T6 transcription when *tfoY* is deleted from C6706 carrying the IGR of V52 may result from indirect effects of TfoY, or a factor(s) specific to V52 and absence in C6706. These results support a hypothesis that a novel CRE lies within the IGR 5′ of the T6 locus, despite a lack of any known direct TF-DNA interactions at this locus identified to date.

10.1128/mbio.00422-22.3 FIG S3 C6706 T6 is no longer activated by QstR after acquiring the G-68T mutation. (A) Competition assays were conducted by co-culturing V. cholerae and Cm^r^ E. coli target cells followed by determination of E. coli survival by counting of CFUs on LB agar with Cm. The V. cholerae *ΔvasK* mutant served as a T6- negative control. (B) Fluorescence levels are from reporters with *gfp* fused to the intergenic region 5’ of *vipA* derived from the strains shown. Data shown are mean values ± S.E. from three independent biological replicates. A one-way ANOVA with Dunnett post-hoc test was conducted to determine the significance. ns, not significant; ****, *P* ≤ 0.0001; ***, *P* ≤ 0.001; **, *P* ≤ 0.01; ns > 0.05. Download FIG S3, TIF file, 1.1 MB. Copyright © 2022 Ng et al. 2022 Ng et al. https://creativecommons.org/licenses/by/4.0/ This content is distributed under the terms of the Creative Commons Attribution 4.0 International license.

To begin mapping the T6 IGR region and SNP locations, we experimentally determined the transcriptional start site (+1) by 5′ rapid amplification of cDNA ends (Materials and Methods). The +1 of transcription resides 320 nucleotides (nt) 5′ of the ATG of the first T6 gene (*vipA*, *vca0107*), and adjacent to a putative promoter with 8/12 identical nucleotides compared with the consensus sigma70-dependent promoter (Fig. 3A). The +1 is consistent with paired-end RNA-seq results we have reported prior (29). Because the majority of 5′ untranslated regions (UTRs) in V. cholerae are 20 to 40 nt, with few exceeding 300 nt (42), we speculate that the 320 nt 5′ UTR of the major T6 gene cluster may be posttranscriptionally regulated, beyond the sRNA interactions already described near the ribosome binding site (RBS) (43). Alignment of the IGRs of C6706 and V52 reveals a single SNP at −68, with a guanine (G) in C6706 at that position and a thymine (T) in V52 (Fig. 3A).

The replacement of the C6706 IGR with V52 was effectively a G-68T mutation (Fig. 3B and C), thus we further tested whether G was necessary for QstR activation by replacing the T with a G at position −68 (T-68G) in the 3223-74 WT, qstR*, and *ΔqstR* backgrounds. The T-68G mutation significantly increases E. coli survival and decreases T6 transcription in WT 3223-74 and the *ΔqstR* derivative, with killing restored in the strain with the qstR* allele (Fig. 3D and E). Thus, a G at position −68 confers inducible, QstR-control, while a T results in constitutive killing *in vitro*, consistent with results recently reported during manuscript revision (44). Based on these results we predicted this SNP is a result of adaptive evolution to control T6 activity in different environments.

### The SNP at −68 is evolutionarily conserved.

**FIG 4:**
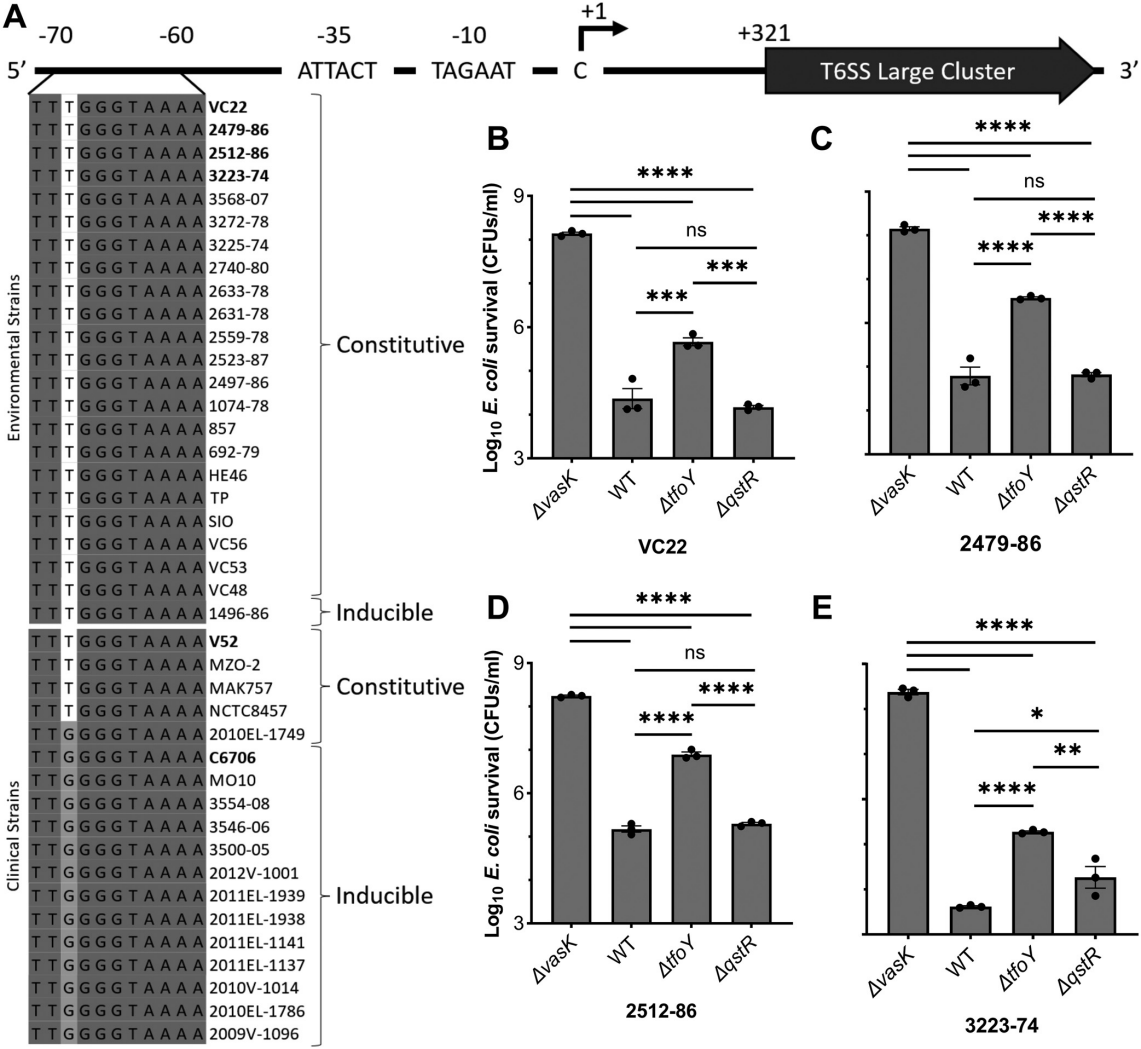
Environmental V. cholerae isolates encode a T at position −68 while human, chitin-induced isolates encode a G. (A) A SNP at position −68 in the IGR of the major T6 cluster controls killing activity. Conserved nucleotides are in dark gray and the SNP of interest is highlighted in white/gray. T6 control was categorized as described (32). (B to E) Survival of E. coli following competition assays with WT V. cholerae strains and mutants was determined by CFU counts. The V. cholerae *ΔvasK* mutant served as a T6- negative control. Data shown are mean values ± S.E. of three independent biological replicates. A one-way ANOVA with Tukey *post hoc* test was conducted to determine the significance. ns, not significant; ****, *P* ≤ 0.0001; ***, *P* ≤ 0.001; **, *P* ≤ 0.01; *, *P* ≤ 0.05.

To determine whether the SNP at −68 is prevalent in V. cholerae, we aligned the T6 IGR sequences of diverse strains that we have characterized prior for T6 killing activity (Fig. 4A) (32). Consistent with prior studies (11, 14, 16, 18), our phylogenetic analysis (Materials and Methods) of the T6 IGRs places human strains in a distinct clade, with the exception of two O1 strains isolated nearly a century ago (NCTC8457 and MAK757), and two non-O1 strains (MZO-2 O14 and V52 O37; Fig. S4). All 23 environmental isolates carry the T-68 SNP and displays constitutive T6 activity, with one exception that is chitin-inducible (1496-86) (Fig. 4A; Fig. S4). By contrast, the 18 human-derived isolates tested carry either G or T at the −68 position (Fig. 4A; Fig. S4). The 13 chitin-inducible human isolates carry a G; five show constitutive activity and carry a T, like environmental strains, with one exception that is constitutive yet carries the G (2010EL-1749) (Fig. 4A; Fig. S4). Neither C nor A are observed at −68 in any stains tested, although both pyrimidine nucleotides (T and C) confer constitutive killing at −68, and both purines (G and A) behave similarly (Fig. S5). The focal SNP location is distal from the promoter, but inconsistent with AT-rich “UP-elements” that reside immediately upstream of the promoter at −38 to −59 and interact directly with the alpha subunit of RNAP (45). We propose the SNP is more likely a component of a CRE for a TF to be determined. Indeed, transversion mutations have greater effects of TF binding than transitions, as noted here (Fig. S5) likely due to changes in shape of the DNA backbone or DNA-amino acid contacts (46, 47).

10.1128/mbio.00422-22.4 FIG S4 Most human isolates are in a clade distinct from environmental isolates. The 6 5’ IGR sequences of the V. cholerae strains described in (32) were used to conduct the maximum likelihood phylogenetic analysis with MEGA. Nag, nonagglutinating; H, human isolates; E, environmental isolates. Download FIG S4, TIF file, 0.4 MB. Copyright © 2022 Ng et al. 2022 Ng et al. https://creativecommons.org/licenses/by/4.0/ This content is distributed under the terms of the Creative Commons Attribution 4.0 International license.

10.1128/mbio.00422-22.5 FIG S5 Transversions at -68 alter T6 control. Transversion mutations (T-68A, T-68G) but not a transition mutation (T-68A) introduced into the 3223-74 IGR change T6 control. Competition assays were conducted by co-culturing V. cholerae with Cm^R^ E. coli at a ratio of 1:10 for 3 h on LB agar plates. Survival E. coli was selected by Cm and determined by counts of CFUs. *ΔvasK* in V. cholerae prevents assembly of T6 and was served as a T6- negative control. Data shown are the mean ± S.E. from three independent biological replicates. A one-way ANOVA with Dunnett post-hoc test was conducted to determine the significance. ns, not significant; ****, *P* ≤ 0.0001. Download FIG S5, TIF file, 0.3 MB. Copyright © 2022 Ng et al. 2022 Ng et al. https://creativecommons.org/licenses/by/4.0/ This content is distributed under the terms of the Creative Commons Attribution 4.0 International license.

We examined regulation of three additional genetically manipulatable environmental strains (VC22, 2479-89, and 2512-86) that exhibit T6 killing (32). Like 3223-74, QstR is expendable in each strain (Fig. 4B to E) while TfoY contributes to some extent in activating T6, with varying E. coli recovery observed in each derivative carrying the *ΔtfoY* mutation (Fig. 4B to E). Taken together, our findings reveal that the constitutive T6 killing activity of environmental V. cholerae is driven by a T at position −68, which obviates the QstR requirement, and permits modest TfoY regulation.

Bacterial adaptation to unexploited niches can be the result of horizontal gene transfer events (5) as well as mutations in protein coding and promoter regions (48, 49). Here we describe an intergenic non coding SNP that coordinates adaptation by altering T6 control between two states—one that is inducible and the other that displays constitutive activity. While the type VI secretion system was first described in V. cholerae in 2006, the knowledge of its regulation remains largely restricted to human isolates, and the identity of a TF that directly controls the major T6 cluster remains elusive to this date (22, 24). We speculate that the focal SNP we identified at position −68 is a component of a CRE that contributes to pathoadaptation (Fig. 3A), a result of adaptive evolution, which allows V. cholerae to carefully control the T6 expression in specific environments. Our results are consistent with the hypothesis that constitutive T6 is beneficial in aquatic environments outside a human host (50), with varying degrees of TfoY contribution, which may act directly or indirectly at the transcriptional or posttranscriptional level (Fig. 3A; Fig. 4B to E; Fig. S6). During human infection where selection promotes dampened T6, V. cholerae with a T-to-G mutation (inducible T6) are favored. In fact, T6-deficient human isolates (e.g., O395) have been reported to have less competitive fitness in human intestinal colonization and infection (19, 51). Although low level, basal expression of T6 contributes to pathogenesis of C6706 (52), overexpression of T6 may be deleterious *in vivo*. Indeed, we have reported prior that V. cholerae with constitutive T6 induces violent peristaltic contractions in a fish host (53), which may disrupt the interaction between V. cholerae and the gut microflora.

10.1128/mbio.00422-22.6 FIG S6 Alignment of T6 IGR of environmental and human-derived isolates. (A) Sequences of the V. cholerae environmental strains described in (32) were collected from NCBI database (Table S4). The T6 5’ IGR sequences were aligned using MUSCLE and generated using ESPript. Conserved bases are highlighted in black, the putative promoter is boxed, and the start codon of *vipA* is in gray. (B) Sequences the V. cholerae human-derived strains described in (32) were collected from NCBI database (Table S4), except 2012V-1001, 2011EL-1939, 2011EL-1938, and 2011EL-1141 that were generated by Sanger sequencing. The T6 5’ IGR sequences were aligned using MUSCLE and generated using ESPript. Conserved bases are highlighted in black, the putative promoter is boxed, and the start codon of *vipA* is in grey. Download FIG S6, TIF file, 2.8 MB. Copyright © 2022 Ng et al. 2022 Ng et al. https://creativecommons.org/licenses/by/4.0/ This content is distributed under the terms of the Creative Commons Attribution 4.0 International license.

There remains a pressing public health need to understand the emergence of pathogens from environmental reservoirs (54). Efforts such as microbial genome wide association studies (55) to identify genetic variants in genomes that are associated with phenotypes like virulence and antibiotic sensitivity will be bolstered by knowledge of the ecological and evolutionary processes that promote pathogen-host association. Defining the plasticity of the regulatory circuity controlling the T6 weapon will provide insights into the role of polymorphisms in the evolution of this and other pathogens.

## MATERIALS AND METHODS

### Bacterial growth conditions and plasmid constructions.

All V. cholerae and E. coli (Table S1) strains were grown aerobically at 37°C overnight in lysogeny broth (LB) with constant shaking or statically on LB agar. Ampicillin (100 μg/mL), kanamycin (50 μg/mL), chloramphenicol (10 μg/mL), spectinomycin (100 μg/mL), streptomycin (5 mg/mL), sucrose (20% wt/vol), and diaminopimelic acid (50 μg/mL) were supplemented where appropriate.

10.1128/mbio.00422-22.7 TABLE S1 List of bacterial strains. Download Table S1, XLSX file, 0.02 MB. Copyright © 2022 Ng et al. 2022 Ng et al. https://creativecommons.org/licenses/by/4.0/ This content is distributed under the terms of the Creative Commons Attribution 4.0 International license.

Plasmids (Table S2) used were constructed with DNA restriction nucleases (Promega, WI, USA), Gibson Assembly mix (New England Biolabs, MA, USA), and PCR amplification (Qiagen, Hilden, Germany) by PCR with Q5 polymerase (New England Biolabs, MA, USA), and primers (Table S3) generated by Eton Bioscience Inc. (NC, USA) or Eurofins Genomics (KY, USA). All reagents were used according to the manufacturer’s instructions. Plasmids were confirmed by PCR and Sanger sequencing by Eton Bioscience Inc. (NC, USA).

10.1128/mbio.00422-22.8 TABLE S2 Plasmid list. Download Table S2, XLSX file, 0.02 MB. Copyright © 2022 Ng et al. 2022 Ng et al. https://creativecommons.org/licenses/by/4.0/ This content is distributed under the terms of the Creative Commons Attribution 4.0 International license.

10.1128/mbio.00422-22.9 TABLE S3 Primer list. Download Table S3, XLSX file, 0.02 MB. Copyright © 2022 Ng et al. 2022 Ng et al. https://creativecommons.org/licenses/by/4.0/ This content is distributed under the terms of the Creative Commons Attribution 4.0 International license.

### V. cholerae mutant construction.

All genetically engineered strains of V. cholerae were constructed with established allelic exchange methods using vector pKAS32 (56) and pRE118 (Addgene - Plasmid #43830). All insertions, deletions, and mutations were confirmed by PCR and Sanger sequencing conducted by Eton Bioscience Inc. (NC, USA). Primers used are in Table S3.

### Fluorescence microscopy.

Variants of V. cholerae strain 3223-74 and an E. coli MG1655 strain with *gfp* introduced into the chromosome were separately back-diluted 1:100 and incubated at 37°C for 3 h. V. cholerae and E. coli were normalized to OD_600_ = 1 and mixed in a 1:5 ratio. A 2 μL aliquot of a mixed culture was inoculated on LB agar and allowed to dry. Cells were imaged before and after a 3 h of incubation at 37°C and 96% to 100% humidity using an Eclipse Ti-E Nikon (NY, USA) inverted microscope with a Perfect Focus System and camera previously described (11). The images were processed with ImageJ (35).

### Motility assay.

Overnight cultures of V. cholerae were diluted to OD_600_ = 0.1, and 1 μL inoculated onto predried LB plates with 0.3% agar. Cells were incubated at 37°C statically overnight, with motility determined by measuring the swarming diameter.

### Transformation assay.

Chitin-induced transformation frequency was measured as described with defined artificial seawater (450 mM NaCl, 10 mM KCl, 9 mM CaCl_2_, 30 mM MgCl_2_·6H_2_O, and 16 mM MgSO_4_·7H_2_O; pH 7.8) (57). Bacteria were incubated with extracellular DNA in triplicate wells containing crab shell tabs, and transformation frequency calculated as Spectinomycin resistant (Spec^r^) CFU mL^−1^/total CFU mL^−1^.

### QS-dependent luciferase assay.

A previously described, pBB1 cosmid was used as a QS-dependent *lux* reporter in V. cholerae (58). Overnight cultures of the V. cholerae strains were diluted to OD_600_ = 0.001 in liquid LB in microtiter plates and incubated at 37°C with shaking. The OD_600_ and luminescence were measured each hour with a BioTek (VT, USA) Synergy H1 microplate reader to calculate relative luminescence units (RLU) as luminescence/OD_600_. V. cholerae without the cosmid served as a negative control (no reporter control). Data were collected when OD_600_ = 0.6 to 0.8. LB medium was used to blank the microplate reader for OD_600_ and luminescence readings.

### Green fluorescent protein gene transcriptional reporter quantification.

Overnight cultures of V. cholerae or E. coli were diluted 1:100 and incubated at 37°C for 3 h. To enhance the translation of *gfp*, the sequence of the native RBS (12 nt sequence) was replaced with the T7 RBS (12 nt sequence) in the primers used to make the fusions. Cm was added to maintain the plasmid-borne versions of reporters that were cloned into plasmid pSLS3. Then, 300 μL aliquots were transferred to black microtiter plates to read the OD_600_ and GFP fluorescence (Excitation: 485, Emission: 528) with a BioTek Synergy H1 microplate reader (VT, USA) to calculate relative fluorescence units (RFU) as fluorescence/OD_600_. LB medium was used as the blank for the OD_600_. Strain lacking reporters were used to blank the spectrophotometer for GFP fluorescence measurements.

### T6-mediated killing assay.

Overnight cultures of V. cholerae or E. coli were back-diluted 1:100 and incubated at 37°C for 3 h. V. cholerae strains and the Cm^r^ E. coli target were normalized to OD_600_ = 1 and then mixed at a ratio of either 10:1 or 1:5. A 50 μL mixed culture was spotted onto LB agar and dried. After a 3 h of incubation at 37°C, cells were resuspended in 5 mL of LB, and serial dilutions were conducted. Finally, the resuspension was inoculated on a LB agar containing Cm to select for the surviving E. coli, which was incubated overnight at 37°C and the E. coli colonies were counted and shown as CFU mL^−1^.

### RNA extraction and determination of the +1 of transcription by 5′-RACE.

Overnight cultures of V. cholerae were back-diluted 1:100 and incubated at 37°C for 3 h before lysing. Three independent cultures of T6-active V. cholerae C6706 qstR* and 3223-74 WT were harvested by centrifugation at room temperature. RNA isolation, genomic DNA removal, and RNA cleanup were performed as previously described (59). Genomic DNA contamination was confirmed by conducting PCR with primer pair specific for 16S rRNA loci (*rrsA*) as previously described (Table S3) (60). RNA purity was confirmed by NanoDrop (260/280 ≈ 2.0).

5′-RACE (Invitrogen, MA, USA) was conducted according to the manufacturer’s protocol with slight modifications. Specifically, SuperScript IV reverse transcriptase (Invitrogen, MA, USA) was used to complete the first strand cDNA synthesis. Two *vipA-*specific primers (GT3056 and GT3060) were used to identify the +1 of transcription for the major T6 gene cluster (Table S3). PCR products were purified with QIAquick PCR purification kit (Qiagen, Hilden, Germany) or Zymoclean gel DNA recovery kit (Zymo Research, CA, USA). Sanger sequencing was conducted by Eton Bioscience Inc. (NC, USA) with the corresponding nesting primer (Table S3).

### Genomic and phylogenetic analysis.

Genome sequences of V. cholerae strains were collected from NCBI Genome database (Table S4) (61). The IGR upstream of major T6 cluster was extracted, aligned, and presented using BLAST+ v2.2.18 (62), MUSCLE v3.8 (https://www.ebi.ac.uk/Tools/msa/muscle/) (63, 64), and ESPript 3.0 (https://espript.ibcp.fr/) (38). The DNA sequence of the IGR was used for phylogenetic analysis, and the phylogenetic tree was constructed by the Maximum likelihood method using MEGA11 (65, 66).

10.1128/mbio.00422-22.10 TABLE S4 Genome list with strains details. Download Table S4, XLSX file, 0.02 MB. Copyright © 2022 Ng et al. 2022 Ng et al. https://creativecommons.org/licenses/by/4.0/ This content is distributed under the terms of the Creative Commons Attribution 4.0 International license.

For 2012V-1001, 2011EL-1939, 2011EL-1938, and 2011EL-1141 that do not have genome sequence available, colony PCR was conducted to amplify the 5′ IGR of the major T6 cluster using OneTaq DNA polymerase (New England Biolabs, MA, USA). PCR products were confirmed with gel electrophoresis and Sanger sequencing by Eton Bioscience Inc. (NC, USA) with the identical primer pair (Table S3).
