# Supplementary figures and images for: Evolution of a cis-Acting SNP That Controls Type VI Secretion in Vibrio cholerae
Source: mBio. 2022 May 23;13(3):e00422-22. doi: 10.1128/mbio.00422-22 (PMC9239110; doi:10.1128/mbio.00422-22)

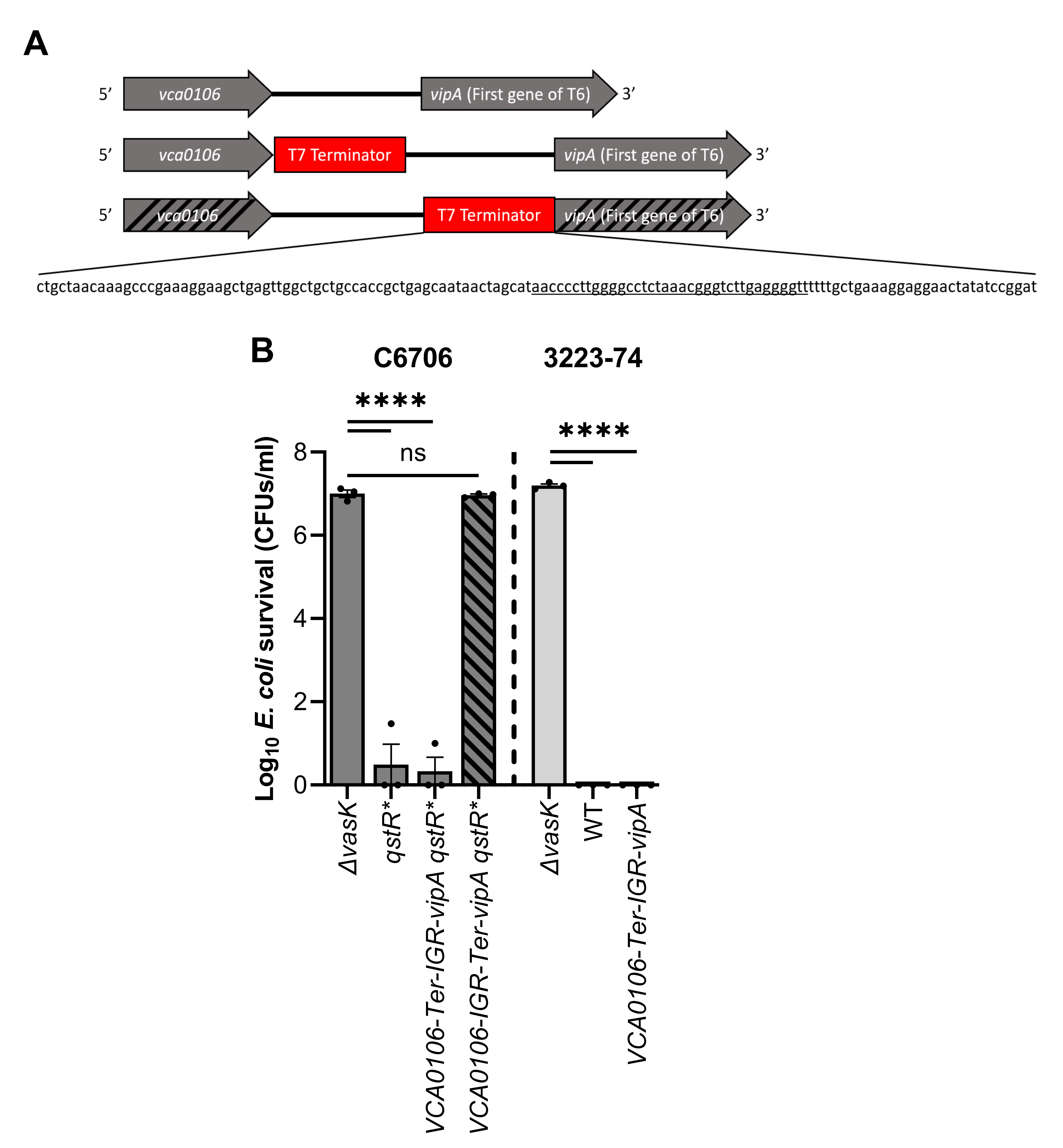

Supplement: FIG S1 [file mbio.00422-22-s0005.tif]

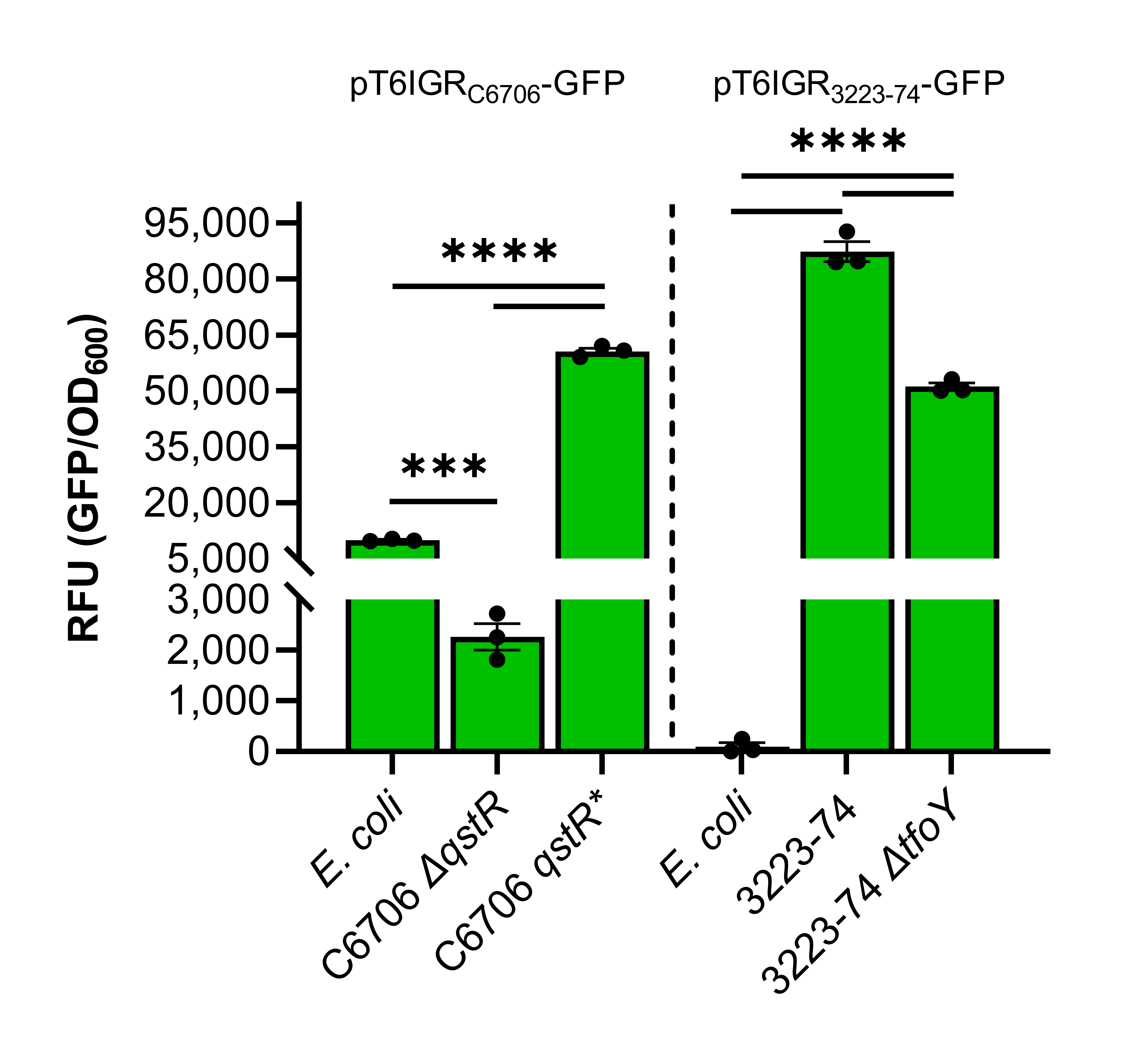

Supplement: FIG S2 [file mbio.00422-22-s0006.tif]

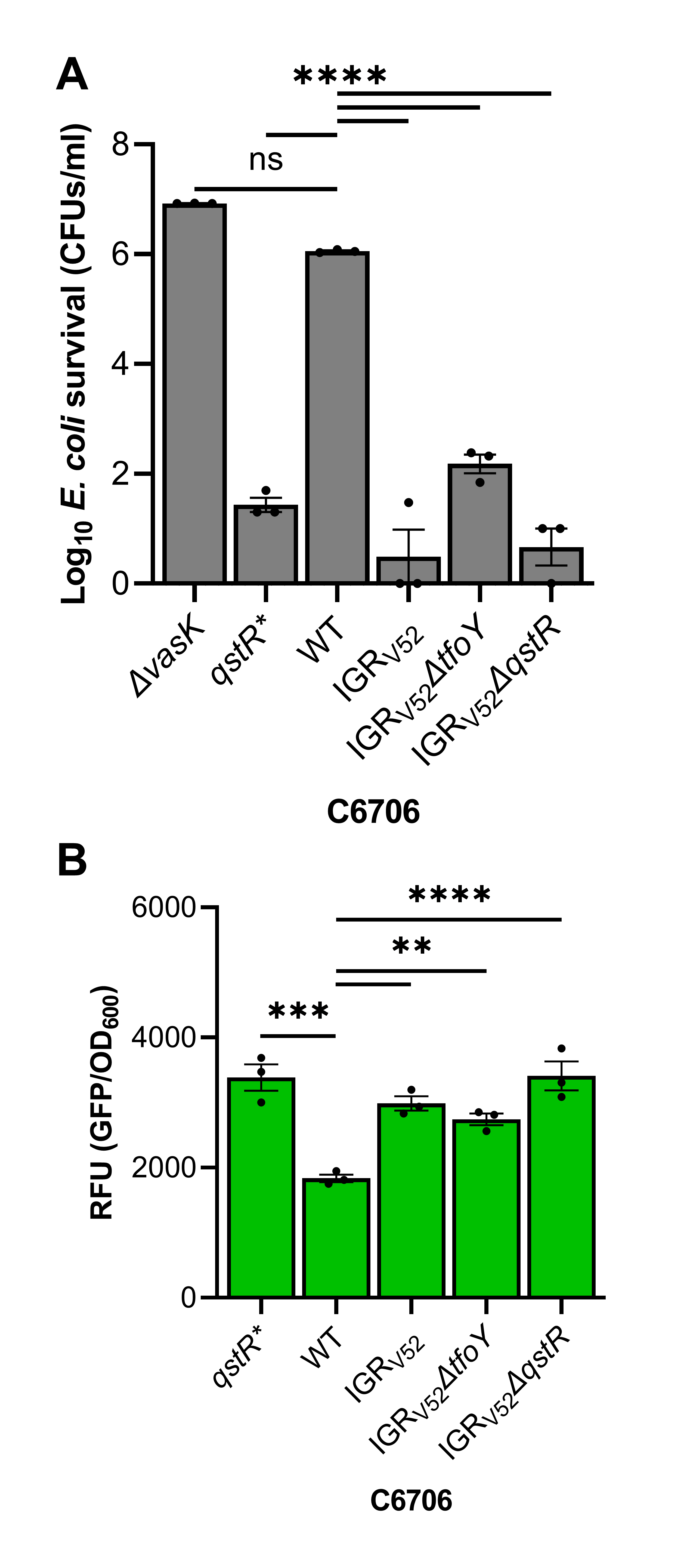

Supplement: FIG S3 [file mbio.00422-22-s0007.tif]

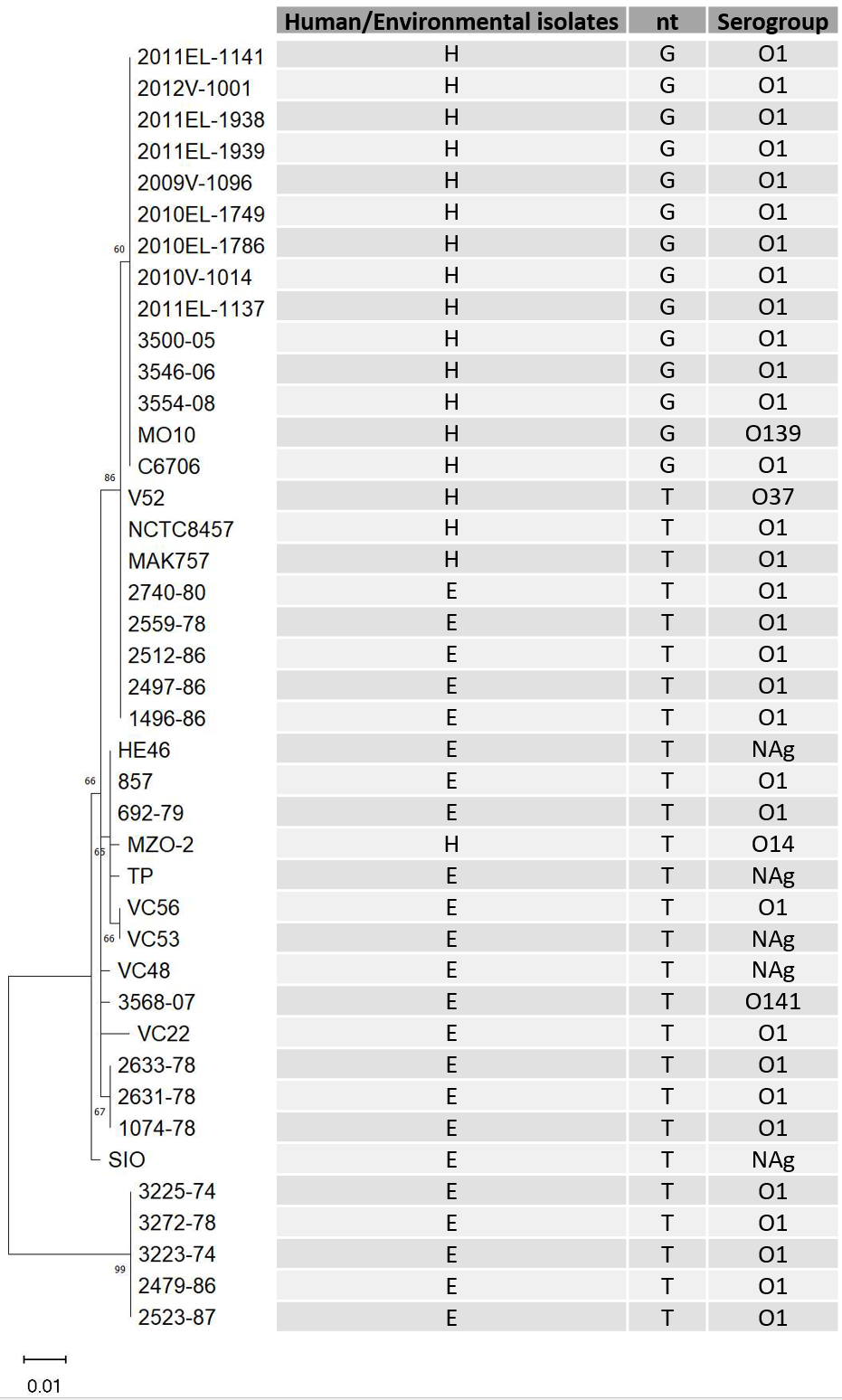

Supplement: FIG S4 [file mbio.00422-22-s0008.tif]

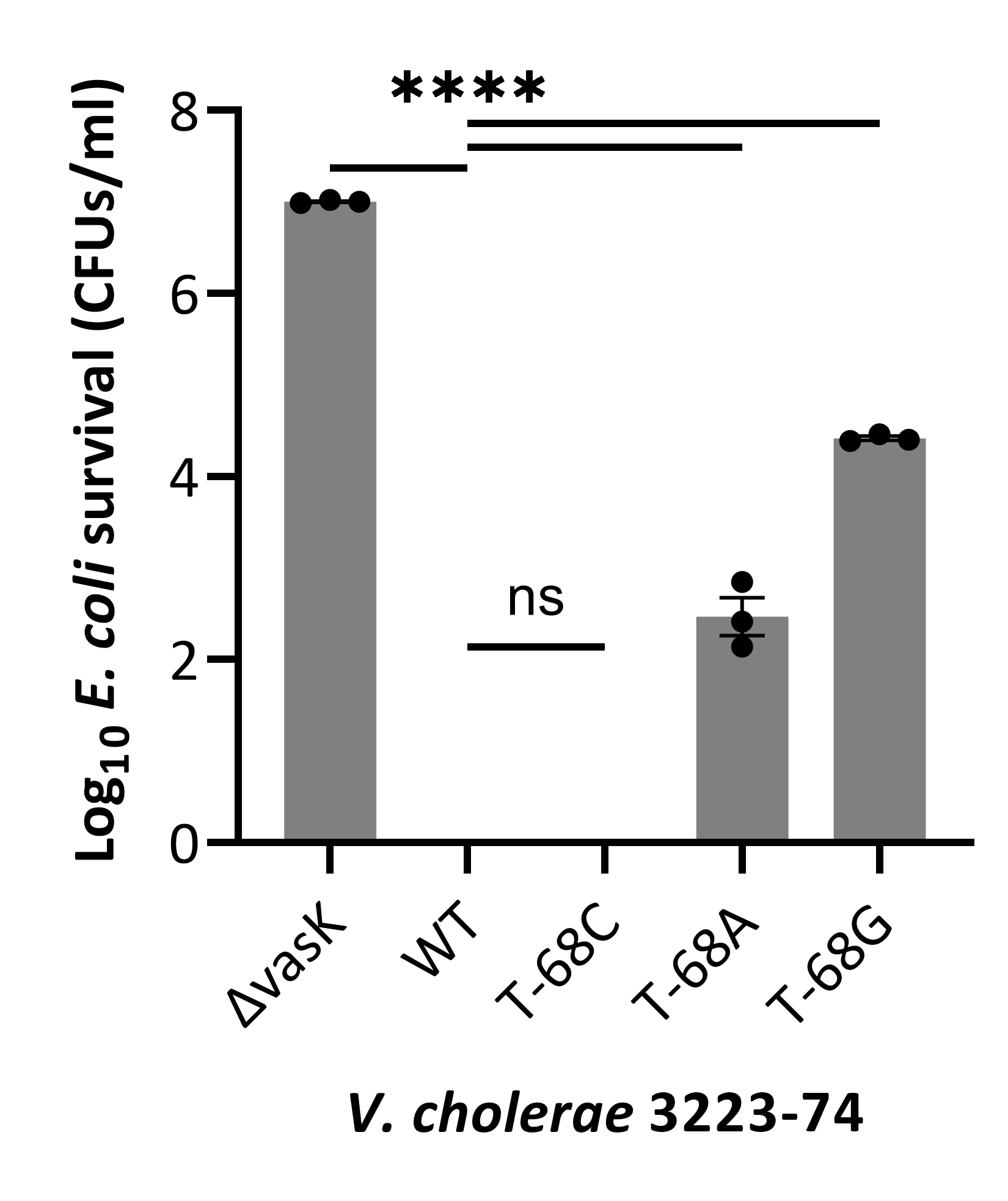

Supplement: FIG S5 [file mbio.00422-22-s0009.tif]

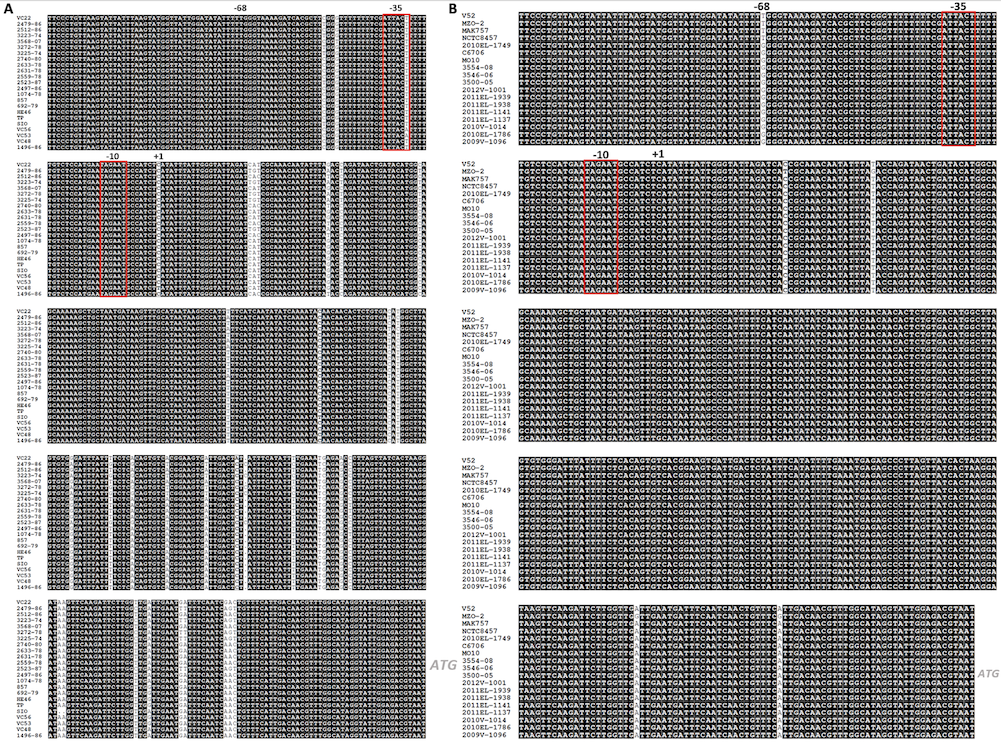

Supplement: FIG S6 [file mbio.00422-22-s0010.tif]
